# Efficacy of Praziquantel in the Treatment of *Platynosomum fastosum* in Cats with Natural Infections

**DOI:** 10.3390/vetsci5020035

**Published:** 2018-03-23

**Authors:** Chele N. Lathroum, Linda Shell, Kathleen Neuville, Jennifer K. Ketzis

**Affiliations:** 1 One Health Center for Zoonoses and Tropical Veterinary Medicine, Ross University School of Veterinary Medicine, Basseterre, St. Kitts, West Indies; chelelathroum@students.rossu.edu; 2 Formerly One Health Center for Zoonoses and Tropical Veterinary Medicine, Ross University School of Veterinary Medicine, Basseterre, St. Kitts, West Indies; linda@vin.com (L.S.); kneuville2@gmail.com (K.N.)

**Keywords:** *Platynosomum fastosum*, liver fluke, praziquantel

## Abstract

Treatments for *Platynosomum fastosum*—the liver fluke of cats—have been developed based on fecal egg counts. Post mortem fluke counts are required to understand true efficacy. In this study, two praziquantel treatment regimens were evaluated using post mortem fluke counts: a high-dose treatment (HT) of 20 mg/kg body weight (BW) administered intramuscularly (IM) once a day for three consecutive days and a low-dose treatment (LT) of 5 mg/kg BW administered once (IM) and repeated 14 days later. A continual enrolment study design was used with 16 naturally infected cats randomly allocated in blocks of four to the HT (eight cats) or LT (eight cats) group. Treatment success, defined as absence of live flukes post mortem, was determined 10 days after the last treatment. Pre- and post-treatment fecal egg counts (centrifugation with Sheather’s sugar flotation solution) and bile egg counts (obtained via percutaneous ultrasound guided cholecystocentesis) were evaluated as supportive efficacy data. Twelve cats completed the study with two cats withdrawn from each group. Neither treatment was 100% effective. In the HT group, three of six cats had live flukes, albeit low numbers, at post mortem, while all six LT group cats had live flukes. While fecal and bile egg counts were reduced in both group, they were not reflective of the true infection status of the cats post mortem.

## 1. Introduction

*Platynosomum fastosum* (syn. *concinnum*, *illiciens* and *planicipitus*) occurs in tropical and subtropical regions including the Caribbean islands, Central and South America, Asia, Australia and the southern United Sates and Hawaii [1]. While infections often are asymptomatic, chronic and heavy infections can result in liver damage with clinical signs including general malaise, jaundice, weight loss, ascites, vomiting and diarrhea [1]. In severe infections, liver failure and ultimately death can occur [1,2,3]. Fecal-based diagnosis can be challenging due to intermittent egg shedding and, in severe cases, due to bile duct blockage preventing egg excretion [2,4].

**Figure 1:**
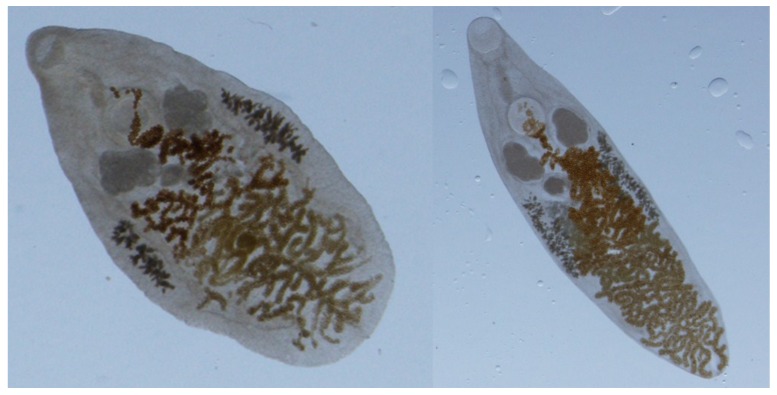
Adult *Platynosomum fastosum* harvested from the liver of a cat.

**Figure 2:**
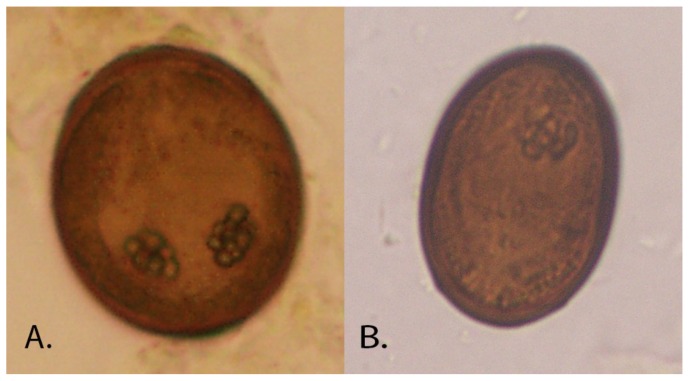
*Platynosomum fastosum* egg in (**A**) bile and (**B**) sugar flotation solution.

The life cycle of *P. fastosum* is complex. Cats become infected by consuming the second intermediate host (terrestrial isopods) or paratenic hosts (insects, toads, lizards and mice) containing metacercaria. A third intermediate host or an obligate paratenic host have been suggested; however, recent work indicates that infection can occur with consumption of the second intermediate host [5]. Metacercariae migrate to the common bile ducts, smaller biliary ducts and gall bladder with mature adults (approximately 1 to 6 mm in length by 0.5 to 2 mm in width; Figure 1) developing within 12 weeks. The eggs (approximately 30 × 50 µm; Figure 2), excreted in cat feces, are consumed by the first intermediate host, land snails (e.g., *Subulina octona*). Daughter sporocysts containing cecaria are then released to the environment where they infect the second intermediate host [1,5]. 

A treatment referenced in the literature for *P. fastosum* infection is praziquantel at 20 mg/kg bodyweight (BW) once daily for three to five consecutive days [1]. This treatment has been extrapolated from studies by Evans and Green (1978) who used a single dose of 20 mg/kg BW and Foley (1994) who used 10 mg/kg BW for three days and recommended a repeat treatment 12 weeks later [6,7]. In both studies, fecal egg counts were used to evaluate efficacy. In a recent case study, a lower dose of praziquantel (5.7 mg/kg BW) administered twice several weeks apart was found to be effective, suggesting that the treatment interval might be as important as the dose [8]. In the study reported here, post mortem fluke counts vs. fecal egg counts were used to assess the efficacy of praziquantel at the dose of 20 mg/kg BW daily for three days. In addition, a lower dose of 5 mg/kg BW (the recommended dose for cestodes and registered dose for praziquantel in cats) administered twice at a 14-day interval was tested. Naturally infected cats were used due to the challenge of inducing *Platynosomum* infections [5].

## 2. Materials and Methods

All applicable international, national, and/or institutional guidelines for the care and use of animals were followed. All procedures performed with the cats were in accordance with the ethical standards of Ross University School of Veterinary Medicine (RUSVM) and conducted under Institutional Animal Care and Use Committee approved protocols (15-1-004, 13-9-017, 15-2-006 and 14-3-009). Sixteen cats were recruited from RUSVM’s Feral Cat Program (FCP) from January to August 2015. The FCP included a feline immunodeficiency virus (FIV) “test and remove policy” in which cats older than 6 months of age positive for FIV (IDEXX SNAP^®^ Feline Triple^®^ Test, IDEXX Laboratories Inc., Westbrook, ME, USA) were euthanized to determine if FIV prevalence (>30%; unpublished FCP data from 2012 to 2013) could be decreased. Cats positive for FIV, with no clinical signs of disease and confirmed positive for *Platynosomum* (using feces or bile) were included in the study. Cats were individually housed, fed a commercial diet at the recommended rate and offered water ad libitum.

A continuous enrolment design was used with cats randomly allocated in blocks of four to either the higher treatment regimen (HT; 20 mg/kg BW once daily for three days) or the lower-dose treatment (LT; 5 mg/kg BW once, repeated two weeks later). Eight cats were allocated to each group, exceeding the minimum of 6 cats recommended for efficacy studies [9]. Post enrolment, one cat was withdrawn from the HT group due to a very low fecal egg count (1 per gram) and no eggs in the bile. Therefore, the HT group included seven cats (all males; 3.67 kg BW, min 2.49, max 4.1 kg at enrollment) and the LT group included eight cats (7 males, 1 female; 3.62 kg BW, min 2.3, max 4.52 kg at enrollment). Table 1 lists the schedule of events.

Praziquantel (Droncit^®^, Bayer, Shawnee Mission, KS, USA) tablets (23 mg and 34.5 mg shaved for exact dosing) were administered on Day 0 to the first cat enrolled in the HT group and the first two cats enrolled in the LT group. Due to the challenge of administering the quantity required in the HT group, 56.8 mg/mL injectable solution ((Droncit^®^ injectable cestocide for dogs and cats) intra-muscularly (IM) administered alternating between the left and right paralumbar muscles) was used for all subsequent treatments for these three cats and for the remaining 12 cats (6 per group).

Fecal egg counts (FECs; double centrifugation with Sheather’s sugar flotation solution; 1–2 g feces) were determined using feces collected from the litter box of individually housed cats or rectum pre-study inclusion and post treatment [4]. Bile egg counts (BECs; direct smear with 40 µL) were determined using bile collected via percutaneous ultrasound guided cholecystocentesis (PUC; performed under anesthesia) prior to treatment and immediately prior to euthanasia. PUC was not performed pretreatment if the veterinarian deemed the procedure a risk to the cat’s health. At least one sample (feces or bile) was required to be *Platynosomum* positive for study inclusion. 

Ten days after the last treatment, the cats were sedated and then euthanized. The liver and gallbladder were removed within 30 min of euthanasia, cut into <2 cm pieces and washed over a 100 µm sieve to collect flukes. Liver and gallbladder pieces were then incubated in saline for a minimum of 3 h (34 to 36 °C) and sieved to collect additional flukes. After collection, flukes were counted, assessed as dead or live based on movement and stored in 10% formalin.

Treatment success, defined as the absence of live flukes at necropsy, was used as the primary assessment of efficacy. All other analyses were considered exploratory with geometrical counts and non-parametric statistical methods used to obtain a more conservative interpretation of results and given the non-normal distribution of helminth egg counts. Secondary analyses included FEC and BEC reductions (FECR; BECR) and HT and LT comparisons. FECR and BECR, for HT and LT, were calculated as: 100 × ((pretreatment geometric mean (GM) FEC − post-treatment GM FEC)/pretreatment GM FEC). The same formula was used for arithmetic means (AM) and for BEC. Increases in FEC and BEC from pretreatment to post treatment were recorded as 0%. If pretreatment FECs and BECs were not significantly different between the two groups (Mann-Whitney test), HT and LT treatment success was compared using the Fisher’s exact test. 

FEC and post mortem fluke count data from nine untreated cats (7 males, 2 females) from the same population but euthanized from July 2014 to February 2015 were available for exploratory analysis to assess the difference in fluke numbers in the HT and LT groups compared to untreated cats. The FEC and fluke data were obtained using the same methods as for the study cats and the FECs were compared between the groups (Kruskal-Wallis test) to confirm that they were not significantly different prior to using the data for calculating the difference in fluke numbers. The formula used was: 100 × ((untreated group GM (AM) fluke count − treated (HT or LT) GM (AM) fluke count)/untreated group GM (AM) fluke count).

## 3. Results

The twelve cats (six per group) administered praziquantel IM were used for the data analysis; cats administered oral praziquantel were excluded from the analysis. While both formulations are registered as cestocides at equivalent doses, trematode efficacy differences are unknown. Three of the six cats in the HT group were negative for live flukes post mortem while all cats in the LT group had live flukes (Table 2). While three cats in the HT group still had flukes, one of these cats only had one fluke and one had two flukes; the remaining cat had 40 flukes. In contrast, one cat in the LT group had 347 flukes with the remainder having 6–25 flukes. 

A pretreatment fecal sample was not collected from one cat (HT group) due to a lack of defecation within the designated time frame. Bile was not collected from two cats (1 HT; 1 LT) pretreatment due to complications (e.g., highly developed mammary glands and anesthesia timeframe limits). Feces and bile were collected from all cats post treatment prior to euthanasia. In the RT group, two cats had no eggs in the feces and bile and no live flukes. One cat that had one live fluke had no eggs in the feces but still had eggs in the bile. Conversely, one cat with no live flukes still had eggs in the feces and bile. In the LT group, three cats with flukes post mortem had no eggs in the feces, one of which also had no eggs in the bile. An additional cat in the LT group had eggs in the feces, but none in the bile.

Pretreatment FECs (N = 5 HT; N = 6 LT) and BECs (N = 5 for HT and LT) were not significantly different between the HT and LT groups (Mann-Whitney *p* = 1.000 and *p* = 0.4034, respectively) allowing comparison of treatment success, which was not significantly different (Fisher’s exact test *p* = 0.0909, one-tailed). The GM FECR and BECR for the HT group (N = 5) was 88% and 99.8%, respectively. The GM FECR and BECR for the LT group was 96.9% (N = 6) and 80.8% (N = 5), respectively. After determining that the FECs of the untreated cats and HT and LT groups were not statistically different (Kruskal-Wallis *p* = 0.996), the exploratory analysis of GM fluke difference was calculated as 99.3% in the HT group and 90.4% in the LT group.

## 4. Discussion

In this study, for the first time, a praziquantel treatment referenced in the literature (20 mg/kg BW for three days) for *P. fastosum* infections in cats was assessed using post-treatment fluke counts. Half of the cats (three of six) were cured of the infection, although one of these was still shedding eggs. The lower dose tested (5 mg/kg BW administered twice at a 14 interval) was not effective in curing the infection, although there was a reduction in FEC and BEC. However, the presence or absence of eggs in feces and bile were not indicative of the infection status post treatment.

Based on the GMs in the exploratory analysis, there was a ≥90% difference in the number of live flukes in the HT and LT groups compared to untreated cats. While this difference suggests that these treatments might reach the minimum requirement of 90% for anthelmintics [9], another study with a parallel control group and blinded fluke counts is required to determine true fluke reduction and efficacy. 

The results of this study with 20 mg/kg BW for three consecutive days are comparable to that achieved by Evans and Green (1978) with a single administration of 20 mg/kg BW [6]. In the Evans and Green (1978) study, five cats were held for three months, allowing for fluke maturation, prior to a single treatment of 20 mg praziquantel/kg BW [6]. Negative fecal samples first occurred approximately 14 days post treatment, although the cats continued to shed eggs intermittently for up to nine weeks. Whether these eggs were from live flukes remaining post treatment or slow clearance of eggs from the bile ducts is not known. However, given the length of time, it is more likely that they were from a few remaining live flukes that eventually died resulting in the negative fecals after nine weeks. These flukes might have been weakened by the treatment, shortening their life span, or died naturally. In the study presented here, euthanasia occurred prior to 14 days allowing less time for eggs to clear from the bile and feces. This resulted in a cat with no flukes to still have a positive fecal result. Hence, egg clearance time must be considered when evaluating efficacy using FEC. It is likely that the two cats with low fluke counts (one and two live flukes), if held for nine or more weeks as in the Evans and Green (1978) study, also would have had intermittent egg shedding and eventually become negative. Foley (1994) suggested that a lower dose (10 mg/kg BW praziquantel) administered on three consecutive days achieved similar results to those of Evans and Green (1978) [6,7]. In the study presented here, consecutive treatments as suggested by Foley (1994) were combined with the high dose used by Evans and Green (1978). This combination is referenced in Basu and Charles (2014). However, the results, given the similarity to that with a single dose, suggest further research is needed to determine if three consecutive treatments, which are challenging to administer, really improve efficacy. Repeating the treatment at a 12 week interval also has been recommended [7]. This would allow any metacercariae consumed near the time of the first treatment to mature before the second treatment. In the study presented here, a two week interval was selected to allow some maturation of immature flukes. While none of the cats were cured, the results indicate that further studies exploring doses lower than 20 mg/kg BW and treatment intervals are warranted. In future studies, a parallel control group as described in the VICH guidelines should be used [9]. In addition, research on the best means of inducing experimental infections would be useful so that efficacy against targeted stages (immature vs. adult flukes) could be evaluated.

## Figures and Tables

**Table 1:** Schedule of events for cats included in the study.

Study Day	Procedures	
≤2 days prior to Study Day 0	Identified for euthanasia by the Feral Cat Program Physical examination by veterinarian Fecal and bile ^1^ examination for *Platynosomum* eggs Body weight ^2^ Allocated to treatment group	
	Group 1	Group 2
Study Day 0 ^3^	Body weight Praziquantel 20 mg/kg BW Clinical assessments	Body weight Praziquantel 5 mg/kg BW Clinical assessments
Study Day 1 ^4^	Praziquantel 20 mg/kg BW Clinical assessments	Clinical assessments
Study Day 2	Praziquantel 20 mg/kg BW Clinical assessments	-
Study Day 7	Clinical assessments	Fecal examination
Study Day 9	Fecal examination	-
Study Day 10	-	Fecal examination
Study Day 12	Fecal and bile examination Euthanasia	-
Study Day 14	-	Body weight Praziquantel 5 mg/kg BW Clinical assessments
Study Day 15	-	Clinical assessments
Study Day 21	-	Fecal examination
Study Day 24	-	Fecal and bile examination Euthanasia

^1^ Bile was obtained using percutaneous ultrasound guided cholecystocentesis. ^2^ Bodyweight was obtained during the physical examination or on Study Day 0 prior to treatment. ^3^ Clinical assessments were performed 30 ± 10 min before and after each treatment and at 3, 8 and 24 h ± 15 min post treatment. ^4^ General health observations (appetite level, fecal and urine output, attitude) were conducted twice daily on non-treatment days.

**Table 2:** Pre- and post-treatment fecal and bile egg counts (FEC; BEC) and calculated reductions.

Group	Flukes		Fecal Egg Counts (Eggs/g)			Bile Egg Counts (Eggs/10 µL)		
	Live Flukes	Percent Difference ^3,5^	Pre-Treatment	Post-Treatment	Percent Reduction ^6^	Pre-Treatment	Post-Treatment	Percent Reduction ^6^
**HT Group ^1^**	**Number of cats (N) = 6**		**N = 5**	**N = 5**	**N = 5**	**N = 5**	**N = 5**	**N = 5**
GM (AM) ^4^ (min, max)	1.5 (7.2) (0, 40)	99.3 (98.3)	45.8 (80.2) (7, 165)	5.5 (32.0) (0, 104)	88.0 (60)	10.4 (33) (2.5, 131)	0.02 (0.5) (0, 1)	99.8 (98.5)
**LT Group ^2^**	**N = 6**	-	**N = 6**	**N = 6**	**N = 6**	**N = 5**	**N = 5**	**N = 5**
GM (AM) (min, max)	19.3 (68.2) (6, 347)	90.4 (84.3)	70.4 (466.5) (9, 2580)	2.2 (11.4) (0, 62.5)	96.9 (97.6)	5.2 (20.4) (0.8, 68.3)	1.0 (4.9) (0, 12.5)	80.8 (76)
**Untreated ^3^**	**N = 9**	-	**N = 9**	-	-	-	-	-
GM (AM)	201.8 (435) (25, 1590)	-	45.6 (145.9) (4, 843.5)	-	-	-	-	-

^1^ HT: high dose praziquantel treatment (20 mg/kg bodyweight for three days administered intramuscularly). ^2^ LT: low-dose praziquantel treatment (5 mg/kg bodyweight once repeated 14 days later). ^3^ Fluke reduction calculations are presented as suggestive but not definitive since the untreated cats were not maintained on study. The untreated cats were from the same source as the study cats but euthanized July 2014 to February 2015 vs. January to August 2015. Fecal egg counts were not significantly different from the pretreatment HT and LT counts (Kruskal-Wallis *p* = 0.962). ^4^ GM (AM): geometric mean and arithmetic mean; in the case of 0, 1 was added to all numbers and then subtracted after calculation of the GM. ^5^ Fluke difference: 100 × ((untreated cat group GM (AM) count − HT or LT group GM (AM) count)/untreated cats GM (AM) count). ^6^ Fecal and bile reduction: 100 × ((individual cat pretreatment − individual cat post treatment)/individual cat pretreatment); The HT and LT groups (all LT cats and IM cats only) were not significantly different in pretreatment fecal and bile egg counts and percent reduction in fecal and bile egg counts (Mann–Whitney, *p* > 0.05).
